# Developing new sugarcane varieties suitable for mechanized production in China: principles, strategies and prospects

**DOI:** 10.3389/fpls.2023.1337144

**Published:** 2024-01-08

**Authors:** Youxiong Que, Qibin Wu, Hua Zhang, Jun Luo, Yuebin Zhang

**Affiliations:** ^1^ National Key Laboratory for Tropical Crop Breeding, Institute of Tropical Bioscience and Biotechnology, Chinese Academy of Tropical Agricultural Sciences, Sanya, China; ^2^ National Key Laboratory for Tropical Crop Breeding, Sugarcane Research Institute, Yunan Academy of Agricultural Sciences, Kaiyuan, China; ^3^ Key Laboratory of Sugarcane Biology and Genetic Breeding, Ministry of Agriculture and Rural Affairs, National Engineering Research Center for Sugarcane, College of Agriculture, Fujian Agriculture and Forestry University, Fuzhou, China

**Keywords:** sugarcane, sugar industry, mechanized production, varieties, prospect

## Abstract

The sugar industry, which relates to people’s livelihood, is strategic and fundamental in the development of agricultural economy. In China, sugar derived from sugarcane accounts for approximately 85% of total sugar production. Mechanization is the “flower” of sugarcane industry. As the saying goes “when there are blooming flowers, there will be sweet honey.” However, due to limitations in land resources, technology, equipment, organization, and management, mechanization throughout the sugarcane production process has not yet brought about the economic benefits that a mechanized system should provide and has not reached an ideal yield through the integration of agricultural machinery and agronomic practice. This paper briefly describes how to initiate the mechanization of Chinese sugarcane production to promote the sound, healthy, and rapid development of the sugarcane industry, and how to ultimately achieve the transformation of sugarcane breeding in China and the modernization of the sugarcane industry from three perspectives, namely, requirements of mechanized production for sugarcane varieties, breeding strategies for selecting new sugarcane varieties suitable for mechanized production, and screening for sugarcane varieties that are suitable for mechanization and diversification in variety distribution or arrangement in China. We also highlight the current challenges surrounding this topic and look forward to its bright prospects.

## Introduction

Sugar industry, which relates to people’s livelihoods, plays a strategic and fundamental role in the development of the national economy. In the “Guidelines on establishing the functional zones for grain food production and the protection areas producing important agricultural products” issued by China’s State Council, raw sugarcane production is listed in the scope of protected areas for producing important agricultural products, which fully demonstrates that the development of the sugarcane industry is related to the national economy and people’s livelihood in China. Generally in China, sugar is primarily derived from sugarcane and sugar beets, with cane sugar accounting for approximately 85% of total sugar production (Chen et al., 2011; Lam et al., 2019; Rajput et al., 2021; Wu et al., 2022a; Xu et al., 2022). In the 2020–2021 crushing season, the total sugar output in China was 10.6666 million tons, with cane sugar contributing 9.134 million tons and accounting for 85.6% of the total sugar output (Zhang et al., 2021).

As the saying goes “when there are blooming flowers, there will be sweet honey.” Sugarcane production, during which mechanization is the “flower”, is a labor-intensive industry. Poorly mechanized production, high labor costs, and diminishing comparative advantages lead to bitterness in the development of the sugarcane industry. Only when there is mechanization throughout the production process, there will be a sweet future for the sugarcane industry. In China, large-scale migration of rural labor to cities, high labor costs, and poor mechanization of sugarcane production restrict the sustainable development of this industry (Lu, 2019; Huang, 2020; Wei and Bei, 2021). Mechanization throughout the production process is not only a fundamental solution to the lack of labor but also an important method for modernizing the technologies used in agricultural production, breaking through yield limitations, and reducing energy consumption. The mechanization of the entire sugarcane production process is a comprehensive project encompassing various aspects including land preparation, ditching, planting, cultivating, weeding, fertilization, pest control, irrigation, harvesting, loading, transportation, ratooning, subsoiling, and smashing sugarcane leaves and returning them to the fields (Ou, 2019). According to the requirements of production at different stages of development, the mechanization of sugarcane production can be reached at different levels to reduce labor intensity, minimize labor costs, improve work efficiency, and achieve overall economic benefits.

In China, mechanization throughout the sugarcane production process is still in its early development stages. Overall, due to limitations in land resources, technology, equipment, organization, and management, this process has not yet brought about the economic benefits that a mechanized system should provide and has not reached an ideal yield through the integration of agricultural machinery and agronomic practice (Zhang et al., 2021; Chen, 2022). From a historical perspective, mechanization is an inevitable trend and a prerequisite for the development of modern agricultural production. The level of mechanization in agricultural industries is a key indicator for international competitiveness and a direct reflection of its capability (Zhang et al., 2021). On a global scale, the improvement of mechanization in production has a significant impact on, or even a great boost to, the scale of sugarcane production and the development of its associated industries (Zhang et al., 2021). The mechanization of sugarcane production is a key focus and a challenging aspect of improving the quality and efficiency of the sugar industry (Zhang et al., 2021). Here, we aim to briefly describe how to initiate the mechanization of Chinese sugarcane production to promote the sound, healthy, and rapid development of the sugarcane industry. Further, we detail how to ultimately achieve the transformation of sugarcane breeding in China and the modernization of the sugarcane industry by addressing three issues, namely, requirements of mechanized production for sugarcane varieties, breeding strategies for selecting sugarcane new varieties suitable for mechanized production, and screening for sugarcane varieties that are suitable for mechanization and diversification in variety arrangement/distribution in China.

## Requirements for sugarcane varieties in mechanized production

**Figure 1:**
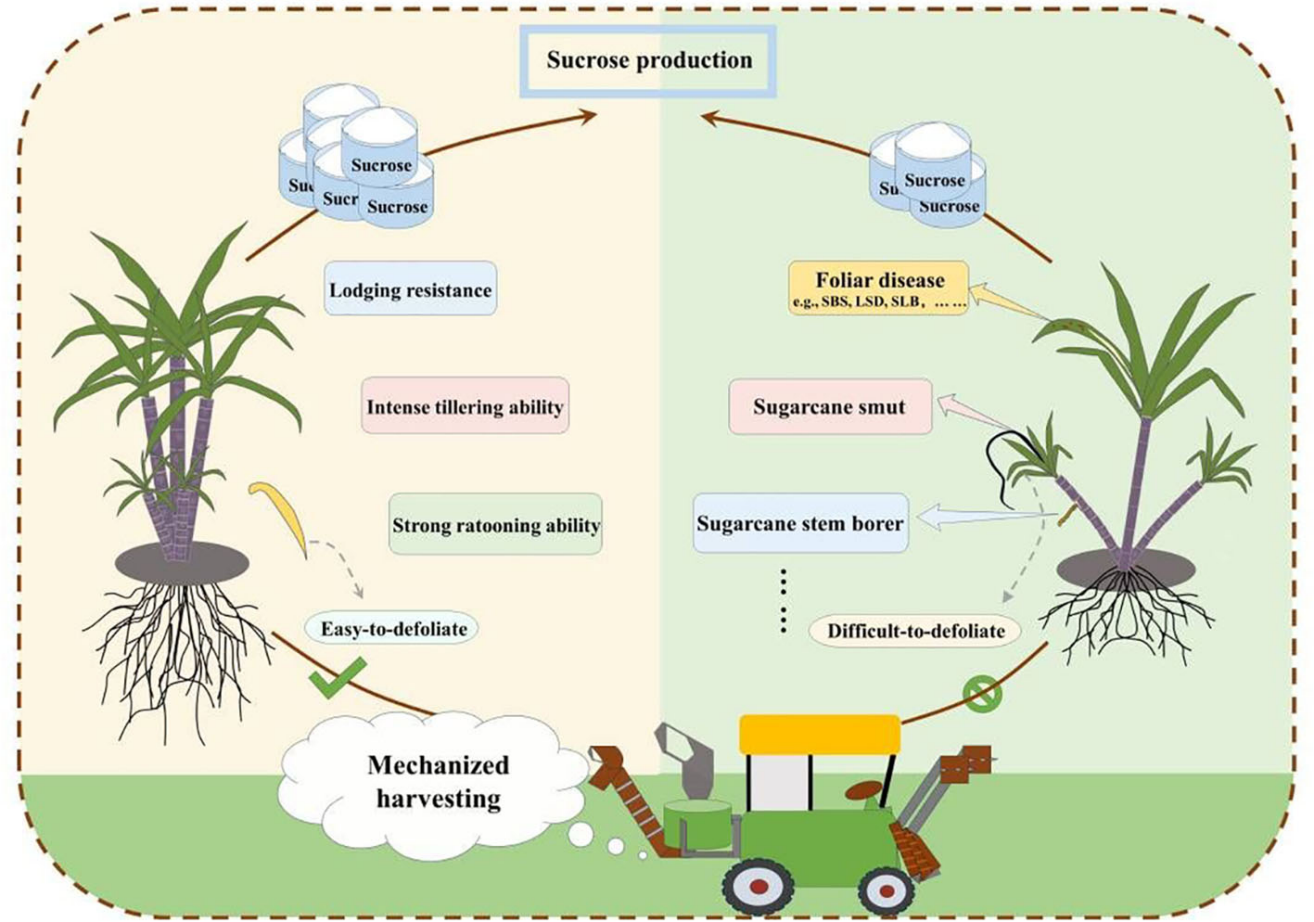
General morphological characteristics of sugarcane varieties suitable for mechanized harvesting. SLB, sugarcane leaf blight; SBS, sugarcane brown stripe; LSD, leaf scald disease.

In sugarcane fields, the efficient and cost-effective utilization of machinery relies on matching crop varieties and agronomic practices (Liu et al., 2023). Existing Chinese sugarcane varieties were mostly selected from hybrid combinations several years or even over a decade ago. During the breeding process, the traits required for mechanical operations have not yet been considered. As a result, in recent years, mechanical harvesting trials and demonstrations have shown that the sugarcane varieties are generally incompatible with mechanical operations. In sugarcane breeding, priority should be given to traits that facilitate improving the efficiency of mechanical operation, reducing impurities in machine-harvested raw sugarcane, minimizing losses caused by machine harvesting, and extending the longevity of the ability to ratoon (Zhang et al., 2021). These traits primarily encompass the following five aspects (**Figure 1**):

(1)High yield, high sugar content, and resistance to locally prevalent diseases are common traits required for new sugarcane varieties. In addition, stalk yield has a direct effect on the efficiency of mechanical harvesting, and thus a higher yield should be considered in breeding.
(2) In terms of phenotypic traits, the new sugarcane varieties should be beneficial for improving harvesting efficiency, reducing harvesting loss, and decreasing the impurity rate in harvested raw sugarcane. It is important that the plants exhibit an upright growth habit, with erect leaves that are easy to detach. The variety should also be resistant to wind and lodging. Plants with few or no aerial roots are mostly welcomed. Varieties that are upright and resistant to lodging may help reduce the rate of stem breakage during mechanical harvesting, ensuring the quality of the harvest (Li et al., 2016; Li et al., 2017; Li, 2019).
(3)The sugarcane variety is required to have the desired growth characteristics, such as quick and even germination, emergence, and tillering, a high rate of stalk formation from tillers, uniform growth rate of the plants, and minimal sprouting in autumn and winter. After planting, robust seedlings with uniform growth can grow as quickly as possible, forming a seedling population with vigorous and even growth, which facilitates inter-row cultivation and helps to improve work efficiency and minimize damage to sugarcane seedlings during mechanical operation (Pierre et al., 2019; Mahadevaiah et al., 2021; Meena et al., 2022; Wang T. et al., 2023).
(4) Maintaining a long-term ratooning ability is an effective measure to reduce sugarcane production costs. During mechanical harvesting, both harvesters and transport vehicles compact the field soil, causing adverse effects on sugarcane ratoons (Xu et al., 2021). This adverse impact is particularly severe when the soil moisture is too high or when the track width of the wheels does not match the row spacing. Selecting varieties with strong ratooning ability is an important procedure that affects the overall effect of mechanical operations in sugarcane fields. To protect sugarcane rows from being directly crushed by the wheels, it is generally necessary to adjust the track width of the wheels or change the planting row spacing so that they can match each other. Sugarcane varieties with strong ratooning ability exhibit several excellent characteristics, such as strong, rapid, and even tillering, which facilitate earlier row closure under a wider row spacing (Gao et al., 2019; Xu et al., 2021; Islam et al., 2023; Wang T. et al., 2023).
(5) Sugarcane varieties suitable for mechanization should possess not only high yield, high sugar content, and resistance to stressors that include diseases, pests, drought, cold, wind, salt, and poor soil, but also strong ratooning ability, bulging stubbles, and deep-set bud eyes. These traits are beneficial for protecting sugarcane buds from mechanical damage. For mechanized tillage management, it is advisable to select sugarcane varieties that exhibit rapid growth in the earlier growth stage, insensitivity to herbicides, strong tillering ability, uniform growth of the main stems and tillers, rapid row closure, resistance to stem breakage, and a high rate of stem formation. Additionally, sugarcane varieties with moderately high fiber content in the stem, the ability to stand upright and resist lodging, easy leaf detachment, loose and thin leaf sheaths, dense cane tissue, and strong sucrose retention and conversion capabilities are suitable for mechanical harvesting (Shang, 2016; Xia et al., 2017; Li, 2019; Yue, 2021).

## Strategies for developing new sugarcane varieties suitable for mechanized production

### Strengthening the breeding of parents and their utilization in hybrid breeding

Sugarcane parents are the material basis for hybrid breeding. To develop excellent new varieties suitable for mechanized operations, it is crucial to start with the breeding of a batch of superior parental materials that possess key characteristics. Therefore, innovative breeding materials, existing parents, materials produced at various stages of hybrid breeding, introduced varieties, and germplasm should be screened for performance and usability in mechanized production and then utilized in further hybrid breeding.

### Adjusting breeding procedures to select offspring based on multiple objectives

With the development of the sugarcane industry, breeding objectives have expanded to consider the breeding of varieties for different uses, such as energy, fruit, and forage sugarcane, in addition to mainstream varieties for producing sugar. In sugarcane hybrid breeding, by making appropriate adjustments to the current breeding procedures and strengthening the selection of relevant target traits during parental and offspring selection, it is possible to achieve diversified breeding objectives within the same breeding program. The breeding of new varieties suitable for mechanical operations can be carried out based on traditional sugarcane breeding procedures, with a focus on the common requirements for mechanical operations, to select varieties with excellent comprehensive traits for further experimental evaluation. Selecting hybrid offspring under fully mechanized operation or wide row spacing helps breed new varieties suitable for mechanical operations. In the early selection stage, based on the primary requirements for mechanical operation, hybrid offspring are selected and subjected to experimental evaluation. This is an important measure for boosting the breeding of varieties suitable for mechanical operations (Zhang et al., 2021).

### Selecting varieties suitable for mechanical operations from existing varieties in production

According to the basic requirements for mechanical operations in sugarcane fields and the characteristics of sugarcane varieties, a batch of new sugarcane varieties can be selected from the existing sugarcane varieties to meet the mechanical operation requirements. In addition, further experiments that combine machinery and agronomic methods can be conducted to screen new varieties suitable for mechanical operation, which should promote the utilization of the varieties and boost mechanization in sugarcane fields (Ou, 2019).

### Application of new technologies in sugarcane breeding

Sugarcane has a complex genetic background, and commercial varieties are highly heterozygous with a genome size of over 10 Gb. It is highly polyploid, resulting from interspecific hybridization between species within the *Saccharum* genus, which limits target gene introgression through backcrossing (Lloyd Evans et al., 2019; Liu et al., 2020; Piperidis and D'Hont, 2020; Trujillo-Montenegro et al., 2021; Ashraf et al., 2022; Shearman et al., 2022). The lack of a high-quality genome sequence for cultivated varieties limits the development of linked markers for useful traits in sugarcane. To date, only markers linked to the *Bru1* gene for brown rust resistance have been widely applied in breeding (Raboin et al., 2006; Wang et al., 2017; Li et al., 2018). Furthermore, although progress is achieved by identifying markers associated with desired traits, there is still a lack of feasible marker-assisted selection technology for breeding. There is still no effective method for the efficient and rapid improvement of sugarcane through biotechnological approaches (Wang et al., 2020; Cheng et al., 2022; Wang et al., 2022; Wu et al., 2022a). Gene engineering has become an important approach to address the shortcomings of traditional sugarcane hybrid breeding and to boost the genetic improvement of this crop. In recent years, there has been increasing research to understand the formation and regulatory mechanisms of important traits in sugarcane, to clone and identify functional sugarcane genes with breeding potential, especially transgenic modifications for sugarcane traits, such as the resistance to insects and diseases (Que et al., 2014; Ling et al., 2022; Ren et al., 2022; Wu et al., 2022b; Sun et al., 2023; Wang D et al., 2023; Wu et al., 2023; Zhao et al., 2023). Notably, substantial progress has also been made in the research and use of gene editing technology in sugarcane, and the safety assessment and commercial application of transgenic sugarcane (Gao et al., 2016; Zhou et al., 2016; Gao et al., 2018; Zhou et al., 2018). It is expected that, there will be an increase in the modernization of sugarcane breeding in the near future.

## Selection of sugarcane varieties suitable for mechanized production and the arrangement of diversified varieties in production

### Selecting varieties suitable for mechanized production is the main direction of sugarcane breeding in the future

Traits suitable for light and simple cultivation operations are essential for enhancing work efficiency and reducing production costs. Adapting to mechanized operations is a key direction for future sugarcane breeding (Zhang et al., 2021). The breeding strategy should be aimed at meeting the requirements of mechanical operation throughout the sugarcane production process. Related traits include those that make mechanical operation and management more convenient, such as lodging resistance and fast tillering, which help achieve relatively consistent heights between the main and tillering stems. In sugarcane production, multiple varieties are used to cope with low temperatures, frost, pests, and diseases. Techniques appropriate for those varieties, such as cultivation, plant protection, integration of mechanical operation and agronomic measures, and seedling techniques, need to be fully considered to obtain a stable yield and balanced increase of output (Zhang et al., 2021).

### Breeding new varieties suitable for mechanical operations in sugarcane production

Through the demonstration of the integrated technical system of the national sugarcane/sugar industry, a large number of varieties with high yield and high sugar content, and are suitable for mechanical operations, can be screened and used in sugarcane-producing areas. The main sugarcane breeding institutes in China include the Sugarcane Research Institute, Guangxi Academy of Agricultural Sciences; Sugarcane Research Institute, Yunnan Academy of Agricultural Sciences; Institute of Nanfan & Seed Industry, Guangdong Academy of Sciences; Sugarcane Research Institute, Fujian Agriculture and Forestry University; Sugarcane Research Institute, Guangxi University; Guangxi Liucheng Sugarcane Research Center; Yunnan Dehong Prefecture Sugarcane Science Research Institute; Sugarcane Research Institute, Fujian Academy of Agricultural Sciences, and Institute of Tropical Bioscience and Biotechnology, Chinese Academy of Tropical Agricultural Sciences. The new sugarcane varieties recommended by each breeding institute can be tested in 15 comprehensive experimental stations within the national sugar industry technical system. Further demonstrations are carried out in 75 counties to select a batch of elite varieties and promising varieties that may play an important role in production. Some varieties, such as LC 05-136, YZ 08-1609, and GT 42, have an average sugar content per hectare exceeding that of ROC22 (Luo et al., 2014; Zhang et al., 2022). They have strong growth, a strong ratooning capability, and good tillering ability and can better adapt to machine-caused soil compaction in the field. Meanwhile, when grown in suitable ecological sugarcane regions, these varieties exhibit a significantly higher yield and sugar content than ROC22. Therefore, by selecting these varieties based on local conditions, it is possible to achieve increased production, higher sugar content, and ultimately, the benefit resulting from the arrangement of diversified varieties in production, which provide strong support for the secure production of sugarcane.

### Rational regional distribution of sugarcane varieties and biplot evaluation of test environments

**Figure 2:**
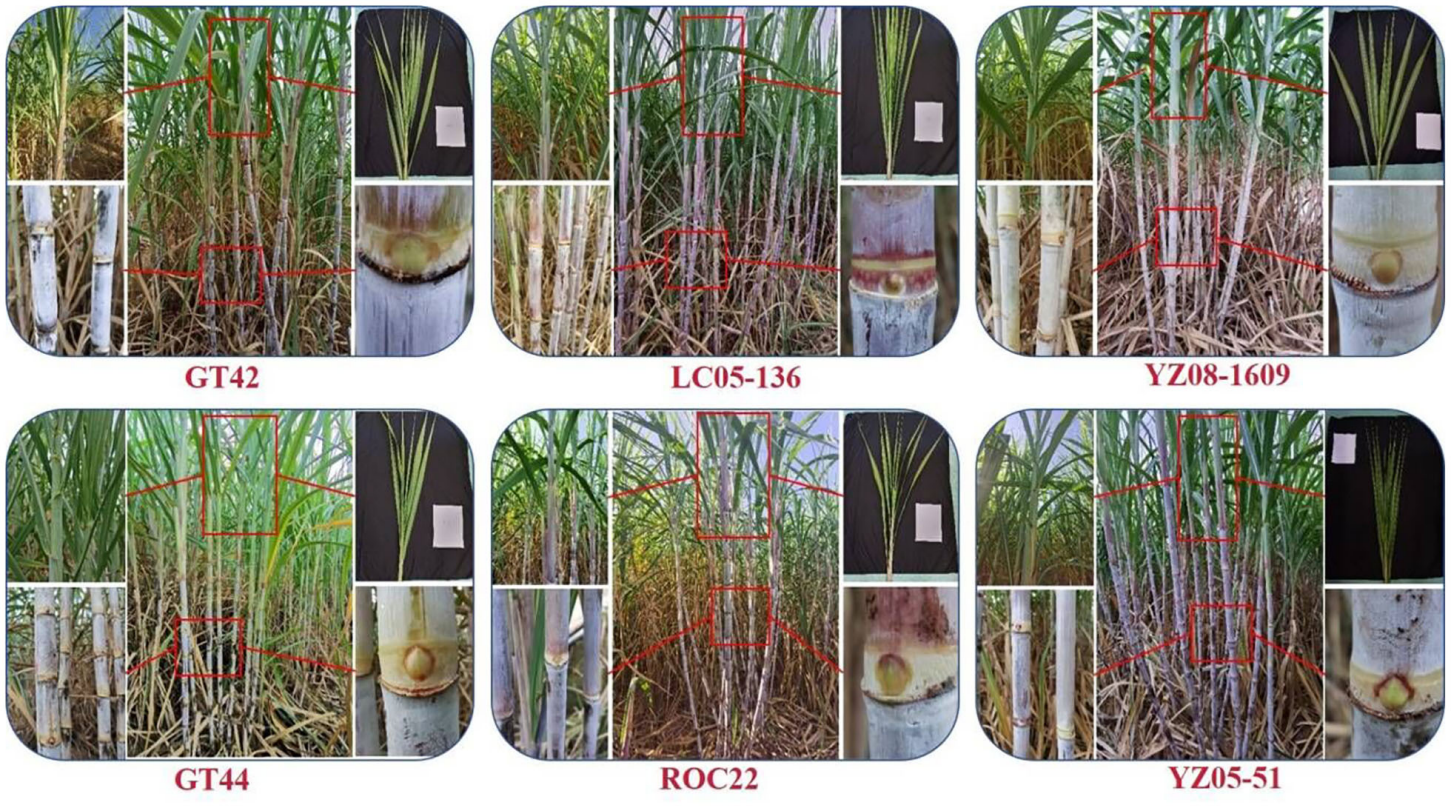
Phenotypic characterization of six major cultivated sugarcane varieties in China.

Regional trials for sugarcane varieties not only allow for the evaluation of the yield potential and stability of candidate varieties, but also facilitate the selection of new sugarcane varieties suitable for specific regions, thereby promoting the diversification or rational distribution of sugarcane varieties in production, which is an important step in the breeding of new sugarcane varieties. Using genotype main effect plus genotype-environment interaction (GGE) and heritability adjusted GGE (HA-GGE) biplot methods, Luo et al. (2015a) selected 18 new sugarcane varieties with both higher sugarcane stalk yield and sugar yield compared with the control variety ROC22. These varieties are recommended for varietal arrangements in sugarcane production according to the local conditions. Among them, the varieties YZ 06-407, FN 39, and YZ 05-51 exhibit strong stability in both sugarcane stalk yield and sugar yield. FN 38, LC 05-136, and FN 1110 show strong stability in sugarcane stalk yield but lower stability in sugar yield. LC 03-1137 and DZ 03-83 display strong stability in sugar yield but lower stability in sugarcane stalk yield. Regarding yield traits, FN 40 and YZ 06-407 have a wider adaptability range and a higher sugarcane stalk yield at multiple trial sites. In addition, Luo et al. (2015b) identified suitable locations and the mega-environments for regional variety trials, further comprehensively evaluated stubble morphology, ratooning ability, and biomass through cluster analysis, and selected sugarcane varieties/lines for production, including DZ 07-36, GT 42, GT 08-2061, and YZ 08-1095, which have high biomass, good ratooning ability, and a well-developed root system. We can reasonably conclude that, all those important varieties with yield and quality potential in the Chinese sugarcane industry, such as LC 05-136, YZ 08-1609, YZ 05-51, GT 42, GT 44, and ROC22, should be comprehensively evaluated to confirm their potential in mechanized production (**Figure 2**).

## Prospects

Sugarcane production in China has reached a historic turning point in achieving mechanization throughout the production process. In recent years, “high-yield and high-sugar” sugarcane production and its protection zones have been established, rural land rights transfer has been implemented, large-scale growers have emerged, and farmland infrastructure suitable for mechanization has been constructed, laying a solid foundation for the mechanization of the sugarcane production process, especially for fully mechanized harvesting. This has also led to the growth of a group of large-scale sugarcane growers and professional service organizations. China’s specific model for sugarcane mechanization is becoming clearer, and the technological roadmap is defined. It has become an industry consensus to simultaneously enhance sugarcane and land productivity under the conditions of high-level mechanization of the production process by breeding varieties suitable for mechanized operations, ensuring that agronomic practices match agricultural machinery operation standards, providing mechanical equipment and technical support for high-yield production, and improving soil structure and soil productivity under the conditions of mechanical operations (Zhang et al., 2021). With the rapid integration of artificial intelligence and big data (Wang X. et al., 2023), research into the mechanization of the entire sugarcane production process is on the rise. We sincerely invite colleagues from home and abroad to work together to speed up the selection and breeding of sugarcane varieties that are suitable for mechanization, resistant to smut, and have a strong ratooning ability. Let all of us involved work together to boost the mechanization of sugarcane production in China to add sweetness to the sugarcane industry.

## Data availability statement

The original contributions presented in the study are included in the article/supplementary material. Further inquiries can be directed to the corresponding authors.

## Ethics statement

Written informed consent was obtained from the individual(s) for the publication of any identifiable images or data included in this article.

## Author contributions

YQ: Conceptualization, Funding acquisition, Project administration, Supervision, Visualization, Writing – review & editing. QW: Formal analysis, Methodology, Software, Visualization, Writing – original draft. HZ: Conceptualization, Resources, Supervision, Writing – review & editing. JL: Conceptualization, Resources, Writing – original draft. YZ: Conceptualization, Funding acquisition, Project administration, Resources, Supervision, Writing – review & editing.
